# [Retracted] Application of a novel targeting nanoparticle contrast agent combined with magnetic resonance imaging in the diagnosis of intraductal papillary mucinous neoplasm

**DOI:** 10.3892/etm.2024.12720

**Published:** 2024-09-18

**Authors:** Min Sun, Liqing Kang, Yanchao Cui, Guoce Li

Exp Ther Med 16:1216–1224, 2018; DOI: 10.3892/etm.2018.6349

Following the publication of this paper, it was drawn to the Editor’s attention by a concerned reader that the RET protein bands featured in the western blots in Fig. 1D on p. 1218 were strikingly similar to the protein data appearing in different form in another article written by different authors at different research institutes that had already been submitted for publication a month in advance of this article to the journal *Molecular Medicine Reports*. In view of the fact that the abovementioned data had already apparently already been submitted for publication previously, the Editor of *Experimental and Therapeutic Medicine* has decided that this paper should be retracted from the Journal. The authors were asked for an explanation to account for these concerns, but the Editorial Office did not receive a reply. The Editor apologizes to the readership for any inconvenience caused.

